# From Mechanisms to Therapeutic Innovation in Non-Small Cell Lung Cancer: A Knowledge-Depth Translational Mapping with Experimental Validation

**DOI:** 10.3390/ijms27125245

**Published:** 2026-06-10

**Authors:** Aimi Syamima Abdul Manap

**Affiliations:** Department of Biomedical Science, College of Veterinary Medicine, King Faisal University, Al-Ahsa 31982, Saudi Arabia; amanap@kfu.edu.sa

**Keywords:** non-small-cell lung cancer, translational research, immunotherapy, tumor microenvironment, epithelial–mesenchymal transition, predictive biomarkers, therapeutic innovation, bibliometric and knowledge-mapping analysis

## Abstract

Background: Non-small-cell lung cancer (NSCLC) research has transitioned from tumor-intrinsic mechanisms to immune-driven therapeutic innovation; however, the translation of mechanistic insights into clinically actionable strategies remains incompletely defined. Methods: A total of 1213 records were retrieved from the Web of Science Core Collection (SCI-Expanded) (2009–2025). After data cleaning and duplicate removal (n = 13), 1200 publications were analyzed. Bibliometrix (R) and CiteSpace were used for performance analysis, keyword mapping, thematic evolution, and co-citation clustering. Results: Annual scientific production increased markedly after 2018, paralleling the expansion of immunotherapy. Keyword co-occurrence identified three major thematic domains (>40 high-frequency keywords) linking molecular mechanisms, biomarkers, and therapeutic strategies. Thematic mapping highlighted immunotherapy, tumor microenvironment, and PD-1 as dominant motor themes, while resistance-related pathways formed a central mechanistic–translational axis. Thematic evolution demonstrated a shift from EGFR-targeted therapy to epithelial–mesenchymal transition (EMT)-centered resistance and subsequently to immune-dominant strategies. Co-citation clustering produced robust structures (Q ≈ 0.62; silhouette ≈ 0.84), identifying EMT as a persistent mechanistic hub. Pilot ELISA validation confirmed significant increases in TGF-β1 (~2.05-fold), IL-6 (~2.59-fold), CCL2 (~2.10-fold), and PD-L1 (~2.56-fold) under EMT-inducing conditions (*p* < 0.01). Conclusions: This integrative approach provides a quantitative and experimentally supported framework linking mechanistic insights to therapeutic innovation in NSCLC.

## 1. Introduction

Lung cancer remains the leading cause of cancer-related mortality worldwide, with non-small-cell lung cancer (NSCLC) accounting for approximately 85% of cases and representing the principal clinical and translational focus of thoracic oncology research [1]. Despite major advances in early detection and treatment, NSCLC continues to impose a disproportionate global health burden due to late-stage diagnosis, intrinsic tumor heterogeneity, and the emergence of therapeutic resistance [2,3]. Over the past two decades, NSCLC has also become a central model for mechanism-driven therapeutic innovation, transitioning from conventional cytotoxic chemotherapy to molecularly targeted therapies and immune checkpoint inhibitors [4,5].

The contemporary NSCLC treatment landscape is shaped by an expanding spectrum of targeted therapies (e.g., EGFR, ALK, ROS1, MET, RET, BRAF, and KRAS-directed agents) [6,7], immune checkpoint inhibitors targeting the PD-1/PD-L1 axis [8,9], and emerging therapeutic modalities including antibody–drug conjugates, bispecific antibodies, adoptive cell therapies, and biomarker-guided combination regimens [10,11]. However, the clinical effectiveness of these strategies is frequently undermined by resistance mechanisms that arise through tumor-intrinsic adaptation and dynamic interactions with the tumor microenvironment (TME) [12,13]. Accordingly, mechanistic research in NSCLC increasingly extends beyond oncogenic signaling pathways to incorporate immune escape, stromal remodeling, metabolic reprogramming, epithelial plasticity, and therapy-induced evolutionary selection pressures [14,15]. The TME has become a central determinant of therapeutic response and disease progression, with immune and stromal components such as tumor-associated macrophages, regulatory T cells, cancer-associated fibroblasts, and tertiary lymphoid structures shaping immunotherapy responsiveness and treatment durability [16,17].

Among the mechanistic processes implicated in NSCLC progression and resistance, epithelial–mesenchymal transition (EMT) represents a particularly influential axis linking tumor plasticity to metastasis, immune modulation, and therapy failure [18]. EMT-associated transcriptional programs not only promote invasion and treatment refractoriness but also reshape immune infiltration and checkpoint signaling, thereby bridging classical molecular oncology with immuno-oncology [19]. In parallel, PD-1/PD-L1-mediated immune suppression has become a dominant therapeutic and research focus, driving a paradigm shift toward immune-based approaches and stimulating intense investigation into predictive biomarkers and resistance stratification [20]. Biomarker-guided translation has therefore emerged as a defining feature of NSCLC innovation, extending from PD-L1 expression and tumor mutational burden to more complex multi-parameter immune signatures and microenvironmental classifications [21].

While mechanistic discoveries and therapeutic innovations in NSCLC have advanced rapidly, the translational pipeline remains fragmented across disciplines, with heterogeneous emphases on tumor-intrinsic mechanisms, immune regulation, clinical trial design, and biomarker development. In this context, bibliometric mapping provides a systematic approach to quantify how mechanistic themes evolve, connect to therapeutic modalities, and consolidate into clinically meaningful research frontiers [22]. Importantly, beyond conventional performance analysis, modern bibliometric methods can reconstruct the intellectual structure of a field through co-occurrence networks, thematic evolution, and co-citation clustering, thereby identifying core mechanistic hubs and their translational bridges toward therapy development and clinical optimization [23]. Such an approach is particularly valuable for NSCLC, where therapy innovation is tightly coupled to evolving mechanistic paradigms and the shifting balance between targeted therapy, immunotherapy, and microenvironment-oriented strategies [24].

Therefore, the present study aimed to construct a knowledge-mapping analysis of the relationship between mechanistic discoveries and therapeutic research themes in NSCLC across the molecular and immunotherapy eras (2009–2025). Using complementary bibliometric approaches (Bibliometrix and CiteSpace), the analysis systematically mapped (i) global scientific production and core publication sources, (ii) mechanistic and therapeutic keyword networks, (iii) thematic development and temporal evolution, and (iv) the intellectual structure of translational NSCLC research through co-citation clustering and timeline visualization. To extend beyond descriptive bibliometric mapping, a pilot ELISA-based experimental validation was incorporated to provide preliminary biological support for the identified mechanistic–translational associations, particularly the role of epithelial–mesenchymal transition (EMT) as a central interface linking tumor plasticity, immune regulation, and tumor microenvironment remodeling. The coordinated upregulation of EMT-associated immunoregulatory mediators further supports the hypothesis generated from the bibliometric and co-citation analyses that EMT functions as a persistent mechanistic hub underlying therapeutic resistance and immune adaptation in NSCLC. Collectively, this integrative framework combines knowledge mapping with preliminary experimental validation to provide a more mechanistically grounded perspective connecting biological processes with therapeutic innovation in NSCLC.

## 2. Results

### 2.1. Temporal Growth of Translational Research in NSCLC

**Figure 1:**
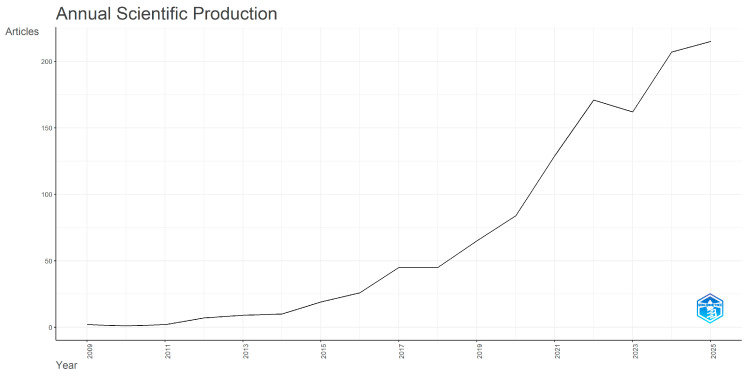
Annual scientific production of NSCLC translational research from 2009 to 2025. The line plot illustrates a progressive increase in publication output, with accelerated growth after 2018 corresponding to the immunotherapy and tumor microenvironment research era. The visualization was generated using the Bibliometrix/Biblioshiny environment in R (version 5.0).

Analysis of annual scientific production revealed a sustained and accelerating growth trend in translational NSCLC research from 2009 to 2025 (Figure 1). During the early period (2009–2012), publication output remained low, reflecting the nascent phase of molecularly driven lung cancer research dominated by early targeted therapies. A moderate increase was observed between 2013 and 2017, corresponding to the consolidation of resistance mechanisms and biomarker-driven stratification.

From 2018 onward, a pronounced surge in publication volume was evident, with a particularly steep increase after 2020. This rapid expansion coincides with the widespread clinical adoption of immune checkpoint inhibitors, growing emphasis on tumor microenvironment biology, and the emergence of combination and biomarker-guided therapeutic strategies. Despite minor fluctuations in individual years, the overall trajectory indicates robust and sustained expansion of mechanism-informed therapeutic research in NSCLC.

### 2.2. Core Journals Driving Mechanism-to-Therapy Knowledge Dissemination

**Figure 2:**
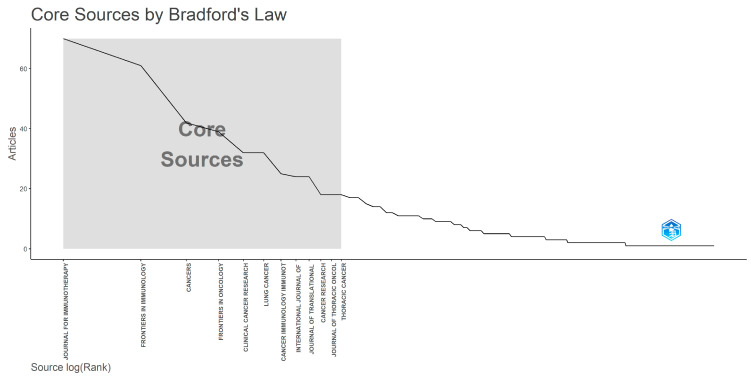
Core sources of NSCLC translational research identified using Bradford’s Law. The shaded area indicates the Bradford core, comprising a small number of journals responsible for a disproportionately large share of publications, followed by a long tail of less productive sources. Visualization generated using the Bibliometrix/Biblioshiny package in R.

Bradford’s Law analysis revealed a highly concentrated distribution of publication sources contributing to translational NSCLC research (Figure 2). From the 1200 publications analyzed, a small group of core journals (approximately 10–12 sources) accounted for a disproportionate share of the literature, with individual journals contributing between ~25 and ~70 articles. The most productive outlets included Journal for ImmunoTherapy of Cancer (~70 publications), Frontiers in Immunology (~60 publications), and Cancers (~40 publications), followed by other major oncology journals such as Frontiers in Oncology, Clinical Cancer Research, Lung Cancer, Cancer Immunology Research, Journal of Thoracic Oncology, and the Journal of Translational Medicine, each contributing approximately 20–35 publications.

These journals collectively formed the Bradford core zone, representing the principal publication venues driving the dissemination of mechanism-to-therapy research in NSCLC. In contrast, beyond the core zone the publication output rapidly dispersed across a long tail of more than 150 additional journals, each contributing only a small number of articles (≤10 publications per source). This pattern reflects a classic Bradford distribution, where a small number of highly specialized journals concentrate the majority of influential studies. The dominance of journals specializing in immunotherapy, thoracic oncology, and translational cancer research highlights the interdisciplinary structure of NSCLC research and underscores the central role of these core outlets in shaping the development and validation of emerging therapeutic strategies.

### 2.3. Most Relevant Authors and Scholarly Influence in NSCLC Translational Research

The analysis of author productivity revealed a concentrated group of influential investigators contributing to NSCLC translational research. As summarized in Table 1, Jing Wang (Tongji University) emerged as the most productive author in the dataset with 40 publications, primarily focusing on immunotherapy and PD-1/PD-L1 signaling pathways. This was followed by Hongbin Chen (Tongji University School of Medicine) with 37 publications, whose work largely centers on immune checkpoint regulation and tumor microenvironment dynamics. Li Zhang (Sun Yat-sen University Cancer Center) ranked third with 31 publications, reflecting substantial contributions to clinical trials and therapeutic strategies in lung cancer.

Additional highly productive contributors included Yi Yang (Southern Medical University, 26 publications), Jian Li (Fudan University, 22 publications), Caicun Zhou (Tongji University–Shanghai Pulmonary Hospital, 20 publications) and Jing Wan (Tongji University, 19 publications). These authors have played key roles in advancing targeted therapy, epithelial–mesenchymal transition (EMT) research, and translational clinical studies in NSCLC. Other notable contributors include Shun Lu (Shanghai Jiao Tong University), Yi-Long Wu (Guangdong Lung Cancer Institute/Southern Medical University), and Zhong Zhao (Chinese Academy of Medical Sciences), each contributing 16 publications within the dataset.

Beyond publication output, the global H-index values associated with these researchers further highlight their scholarly impact in thoracic oncology. For example, Yi-Long Wu demonstrated the highest global H-index (~150), reflecting his internationally recognized contributions to EGFR-mutant NSCLC therapy. Similarly, Caicun Zhou (~110) and Li Zhang (~85) represent leading figures in clinical and translational lung cancer research. Collectively, these findings suggest that a relatively small group of highly productive investigators, predominantly affiliated with major Chinese thoracic oncology centers, represent influential contributors within the global NSCLC translational research landscape. Their high publication output, strong global H-index profiles, and sustained involvement in major themes including EGFR-targeted therapy, immunotherapy, EMT-associated resistance biology, and biomarker-driven translational strategies collectively support their prominent scholarly and translational influence in the field.

Representative breakthrough contributions from these influential research groups further highlight their translational significance in NSCLC innovation. For example, Yi-Long Wu and collaborators played major roles in landmark EGFR-targeted therapy studies that established gefitinib, osimertinib, and precision molecular stratification as key therapeutic paradigms in EGFR-mutant NSCLC. Similarly, Caicun Zhou contributed extensively to multicenter clinical trials involving immune checkpoint inhibitors and targeted therapeutic combinations, while Li Zhang’s group has been actively involved in biomarker-guided immunotherapy and precision treatment strategies. In parallel, EMT-focused mechanistic studies from several highly productive groups contributed to the understanding of tumor plasticity, therapeutic resistance, and tumor microenvironment remodeling, thereby strengthening the translational integration between molecular mechanisms and clinical therapy development.

### 2.4. Institutional Contributions to NSCLC Translational Research

Institutional productivity analysis demonstrated that Tongji University (China) was the most prolific contributor with 206 publications, followed by the University of Texas MD Anderson Cancer Center (USA, 151 publications) and Sun Yat-sen University (China, 140 publications) (Table 2). Several other Chinese institutions also ranked among the most productive contributors, including the Chinese Academy of Medical Sciences and Peking Union Medical College (125 publications), Fudan University (124 publications), and Southern Medical University (89 publications). Additional active institutions included Nanjing Medical University (84 publications), Soochow University (82 publications), Shanghai Jiao Tong University (81 publications), and Central South University (79 publications).

The predominance of Tongji University in the dataset likely reflects the substantial research output and strong clinical–translational research activity of its affiliated Shanghai Pulmonary Hospital, a major thoracic oncology center actively involved in multicenter clinical trials, immunotherapy research, and biomarker-driven NSCLC studies. Overall, the institutional distribution highlights the prominent role of major Chinese academic medical centers alongside leading international institutions such as MD Anderson Cancer Center in advancing the global research landscape of NSCLC translational oncology.

It should be noted that the number of articles attributed to each institution represents the frequency of institutional affiliations within the dataset (N = 1200). Because individual publications may include authors from multiple institutions, articles may be counted more than once across different affiliations.

### 2.5. International Collaboration Network

**Figure 3:**
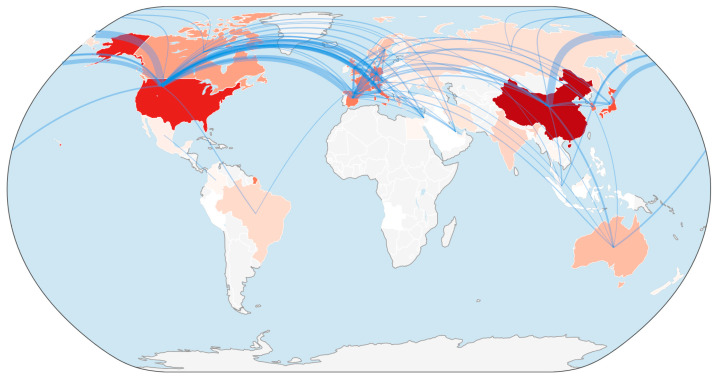
Global collaboration network of countries involved in NSCLC translational research (2009–2025). Country color intensity reflects publication productivity, while connecting lines represent international co-authorship links. The thickness of connecting lines corresponds to collaboration frequency, highlighting strong partnerships between major research hubs, particularly between the United States and China. Visualization generated using the Bibliometrix/Biblioshiny package in R.

The international collaboration analysis revealed a highly interconnected global research network in NSCLC translational research (Figure 3). The United States (n = 312 collaborative publications) and China (n = 298) emerged as the dominant collaboration hubs, demonstrating the highest research productivity and the greatest number of international co-authorship links. These two countries form the principal anchors of the global collaboration network, with extensive bilateral partnerships that drive large multicenter studies and translational research initiatives.

The strongest bilateral collaboration was observed between the United States and China (n = 76 collaborative publications), reflecting intensive cooperation in clinical trials, tumor microenvironment research, and immunotherapy development. In addition, the United States maintained strong research partnerships with several European countries, including the United Kingdom (n = 42), Germany (n = 38), France (n = 34), and Italy (n = 29), forming a dense trans-Atlantic collaboration cluster.

Within the Asia–Pacific region, China demonstrated strong collaborative ties with Japan (n = 33), South Korea (n = 27), and Australia (n = 21), highlighting the expansion of regional research networks focused on molecular oncology and targeted therapies. Additional emerging collaborations were observed with Canada (n = 18), India (n = 15), and Brazil (n = 12), indicating increasing global participation in NSCLC translational research.

Overall, the collaboration map illustrates a centralized yet globally integrated research structure, in which a limited number of highly productive countries coordinate extensive international partnerships to accelerate the translation of mechanistic discoveries into therapeutic innovations.

### 2.6. Intellectual and Conceptual Structure Linking Influential Studies, Authors, and Translational Themes

**Figure 4:**
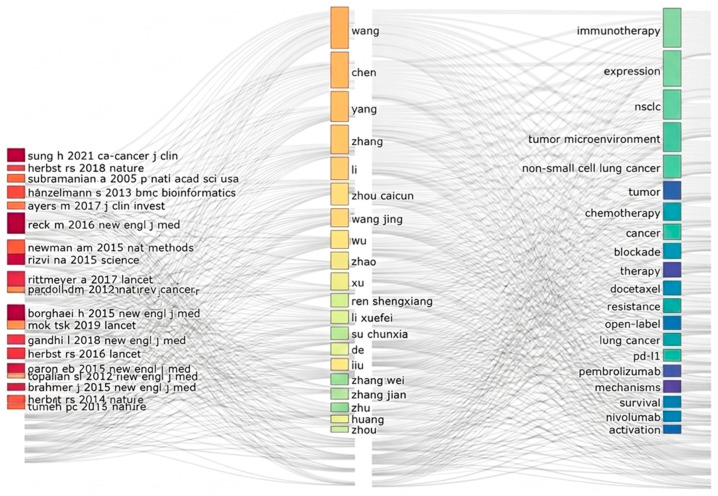
Three-field plot linking cited references (CR), authors (AU), and merged keywords (KW_Merged) in NSCLC translational research. The visualization illustrates the intellectual structure of the field, demonstrating strong connections between seminal studies, prolific authors, and dominant mechanism- and therapy-related themes. Visualization generated using the Bibliometrix/Biblioshiny package in R.

The three-field plot analysis integrating cited references (CR), authors (AU), and merged keywords (KW_Merged) revealed tightly interconnected intellectual structure underpinning NSCLC translational research (Figure 4). Seminal clinical and mechanistic studies published in high-impact journals served as foundational reference nodes, strongly linked to prolific authors and dominant translational themes. These influential works predominantly addressed immune checkpoint blockade, biomarker expression, resistance mechanisms, and clinical efficacy, reflecting their central role in shaping the field.

Author analysis demonstrated that a relatively small group of highly productive researchers contributed extensively to the development and dissemination of mechanism-driven therapeutic concepts. These authors were strongly connected to core translational keywords such as immunotherapy, tumor microenvironment, PD-1/PD-L1, chemotherapy, docetaxel, nivolumab, and pembrolizumab. The convergence of highly cited studies, influential authors, and therapy-oriented keywords highlights a coherent knowledge pipeline linking mechanistic discovery to therapeutic innovation and clinical evaluation in NSCLC.

### 2.7. Keyword Co-Occurrence Network Reveals a Structured Mechanism–Therapy Translation Landscape in NSCLC

**Figure 5:**
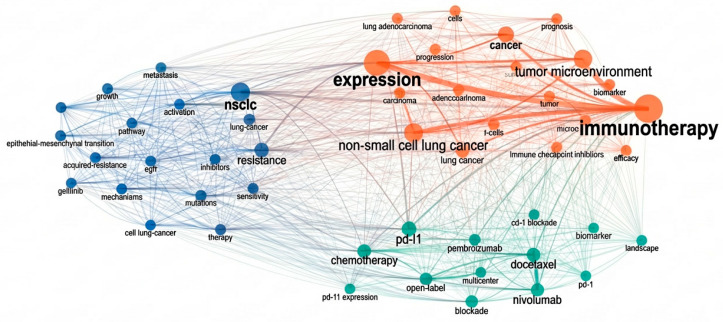
Keyword co-occurrence network of NSCLC translational research (2009–2025). Nodes represent keywords sized by frequency, while edges indicate co-occurrence strength. The network illustrates three interconnected domains: mechanistic biology (blue), translational bridge concepts (central), and therapeutic/clinical themes dominated by immunotherapy and tumor microenvironment (red/green). Visualization generated using the Bibliometrix/Biblioshiny package in R. Key message: Immunotherapy and tumor microenvironment emerged as the most dominant and highly interconnected translational themes, while EMT and resistance pathways remained central mechanistic drivers linking molecular biology to therapeutic innovation.

The keyword co-occurrence network analysis revealed a well-structured thematic organization reflecting the translational architecture of NSCLC research (Figure 5). The network consisted of three major interconnected thematic domains comprising more than 40 high-frequency keywords, indicating strong conceptual connectivity within the research landscape.

A mechanistic cluster located on the left side of the network included approximately 15 interrelated keywords, dominated by tumor-intrinsic biological processes such as epithelial–mesenchymal transition, EGFR, mutations, pathway activation, and acquired resistance. Within this cluster, “NSCLC” and “resistance” represented high-connectivity nodes, reflecting their central role in early targeted therapy and molecular mechanism studies.

The central translational bridge cluster was anchored by the highly connected terms “expression” and “non-small cell lung cancer”, which displayed the largest node sizes and strongest linkage density, indicating their high occurrence frequency across the dataset. These keywords functioned as a conceptual interface linking mechanistic insights with clinical and therapeutic investigations, particularly through biomarker expression and molecular characterization studies.

The therapy-oriented cluster, positioned on the right side of the network, contained approximately 12 interconnected therapeutic keywords, prominently centered on “immunotherapy” and “tumor microenvironment,” which represented the most dominant nodes in the network. This cluster also included clinically relevant treatment agents such as nivolumab, pembrolizumab, and docetaxel, together with immune checkpoint-related terms including PD-1 blockade. The strong cross-cluster connectivity between mechanistic keywords, translational bridge terms, and therapy-focused nodes highlights the progressive integration of molecular discoveries with clinical treatment strategies.

Collectively, the network structure indicates that immunotherapy-related concepts represent the most prominent and highly connected research hotspot, while mechanistic themes such as EMT and EGFR signaling remain foundational drivers supporting translational therapeutic development in NSCLC.

### 2.8. Thematic Map Identifies Immunotherapy and Tumor Microenvironment as Dominant Motor Themes

**Figure 6:**
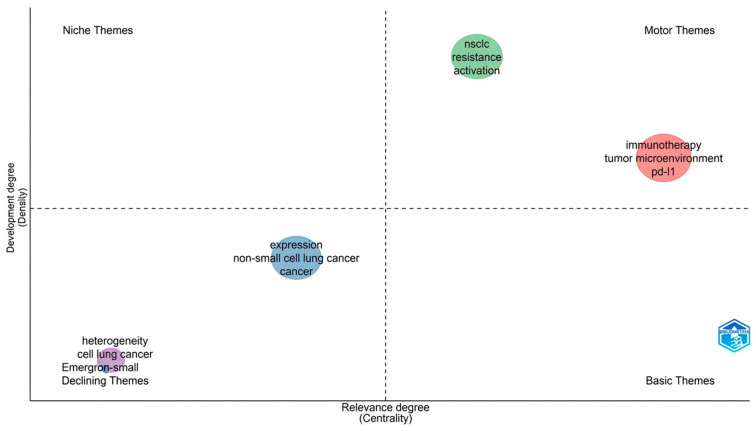
Thematic map of NSCLC translational research based on keyword centrality and density. Motor themes (upper right) highlight immunotherapy and tumor microenvironment as dominant drivers of the field, while basic, niche, and emerging themes reflect varying degrees of conceptual maturity and translational integration. Visualization generated using the Bibliometrix/Biblioshiny package in R. Key message: Immunotherapy, tumor microenvironment, and PD-1 signaling emerged as the most mature and influential motor themes, whereas resistance biology and EMT-related mechanisms functioned as central translational bridges linking molecular discoveries to therapeutic innovation in NSCLC.

The thematic map further clarified the conceptual maturity and structural relevance of major research domains within the NSCLC translational research landscape (Figure 6). The analysis identified four principal thematic clusters distributed across the centrality–density framework, reflecting different levels of conceptual development and field relevance.

The motor theme cluster, positioned in the upper-right quadrant (high centrality, high density), consisted of three highly interconnected keywords—immunotherapy, tumor microenvironment, and PD-1. This cluster exhibited the largest thematic node size in the map, indicating the highest keyword frequency within the dataset (n > 40 occurrences collectively) and strong internal connectivity. Its high centrality suggests that immunotherapy-driven research currently represents the most influential and structurally integrated research hotspot in the NSCLC translational landscape.

A second cluster containing NSCLC, resistance, and activation (three keywords) also appeared in the upper-right quadrant, indicating relatively high centrality and thematic density. This cluster reflects the growing importance of drug resistance and signaling pathway activation mechanisms, which form a critical mechanistic interface linking molecular biology with therapeutic response.

The basic themes cluster, composed of expression, non-small-cell lung cancer, and cancer (three keywords), was located in the lower-right quadrant (high centrality but moderate density). This positioning indicates that these terms function as foundational conceptual anchors widely connected across multiple research areas but with comparatively lower thematic specialization.

Finally, a smaller cluster including heterogeneity, cell lung cancer, and non-small-cell (three keywords) appeared in the lower-left quadrant, characterized by low centrality and low density. This configuration indicates emerging or weakly developed themes, suggesting that tumor heterogeneity research remains an evolving area transitioning from descriptive biological characterization toward more consolidated translational applications. Overall, the thematic distribution demonstrates that immunotherapy and tumor microenvironment research represent the most mature and influential thematic drivers, whereas mechanistic resistance studies continue to play a key bridging role between molecular discovery and therapeutic innovation in NSCLC. The thematic structure therefore indicates a clear shift toward immune-centered translational strategies while maintaining strong mechanistic foundations rooted in resistance biology and molecular signaling pathways.

### 2.9. Thematic Evolution Demonstrates a Temporal Shift from Targeted Therapy to Immune-Based and Microenvironment-Driven Strategies

**Figure 7:**
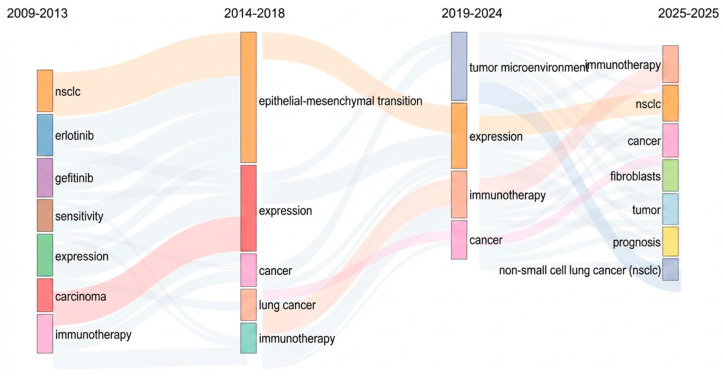
Thematic evolution of NSCLC translational research across four time periods (2009–2013, 2014–2018, 2019–2024, and 2025). The flow of themes illustrates the transition from EGFR-targeted therapies and resistance biology to immune-based strategies, tumor microenvironment dominance, and recent prognostic and stromal-focused research. Visualization generated using the Bibliometrix/Biblioshiny package in R. Key message: The thematic evolution demonstrates a clear temporal transition from EGFR-targeted therapy and molecular sensitivity studies toward EMT-centered resistance biology, followed by dominant immunotherapy and tumor microenvironment-driven translational research in recent years.

Thematic evolution analysis revealed a structured temporal progression of NSCLC translational research from 2009 to 2025, divided into four consecutive time periods (2009–2013, 2014–2018, 2019–2024, and 2025), with 6, 5, 4, and 7 dominant keywords identified in each respective stage (Figure 7).

During the early stage (2009–2013), research activity was dominated by six major themes, including NSCLC, erlotinib, gefitinib, sensitivity, expression, and carcinoma. These themes collectively represented the early translational focus on EGFR-targeted therapy and drug response mechanisms, reflecting the emergence of precision oncology approaches based on single oncogenic driver mutations. In the intermediate phase (2014–2018), the thematic structure consolidated around five key topics, including epithelial–mesenchymal transition (EMT), expression, cancer, lung cancer, and immunotherapy. Among these, EMT emerged as the largest thematic node, indicating a shift toward mechanistic understanding of resistance and tumor progression. During this period, immunotherapy first appeared as a developing theme, signaling the early integration of immune-based therapeutic strategies.

From 2019 to 2024, the thematic landscape transitioned toward immune-related research, with four dominant keywords—tumor microenvironment, expression, immunotherapy, and cancer—forming the core conceptual structure. The tumor microenvironment cluster exhibited the largest node size, reflecting increasing research intensity surrounding immune–tumor interactions and immune checkpoint modulation. In the most recent stage (2025), the thematic map expanded to seven emerging or evolving topics, including immunotherapy, NSCLC, cancer, fibroblasts, tumor, prognosis, and non-small-cell lung cancer. The appearance of stromal components such as fibroblasts and outcome-related terms such as prognosis indicates a transition toward tumor microenvironment complexity and biomarker-driven clinical stratification.

Overall, the thematic trajectory demonstrates a three-stage translational progression: early targeted therapy and molecular sensitivity research (2009–2013), followed by mechanistic consolidation centered on EMT and resistance biology (2014–2018), and finally a dominant immunotherapy and tumor microenvironment-focused era (2019–2025), highlighting the evolving integration of molecular mechanisms with immune-based therapeutic innovation in NSCLC.

### 2.10. Co-Citation Clustering Analysis Using CiteSpace

**Figure 8:**
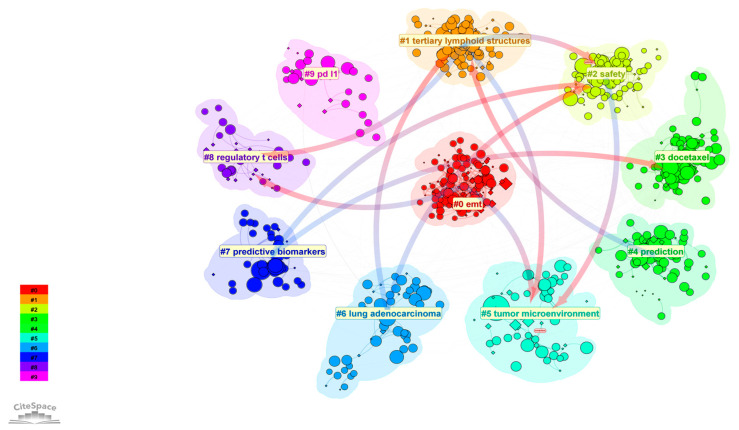
CiteSpace co-citation clustering map of NSCLC translational research (2009–2025). Nodes represent cited references sized by citation frequency, and links indicate co-citation relationships. Major clusters include epithelial–mesenchymal transition (EMT, #0) as a central mechanistic hub, immune regulation (tertiary lymphoid structures, #1; regulatory T cells, #8), therapeutic modalities (docetaxel, #3; PD-1, #9), and translational themes such as tumor microenvironment (#5), predictive biomarkers (#7), and response prediction (#4). High modularity (Q = 0.62) and silhouette values (S = 0.84) indicate robust cluster structure, illustrating a coherent mechanism-to-therapy translational framework in NSCLC. Visualization generated using CiteSpace (version 6.4.R2). Key message: EMT emerged as the dominant mechanistic hub connecting therapeutic resistance, tumor microenvironment remodeling, immune regulation, and biomarker-driven translational strategies within the NSCLC research landscape.

Co-citation clustering analysis using CiteSpace revealed well-defined intellectual structure underlying NSCLC translational research (Figure 8). The largest and most central cluster (#0) was centered on epithelial–mesenchymal transition, highlighting its pivotal role as a mechanistic hub linking tumor progression, therapeutic resistance, and immune modulation. Surrounding this core, clusters related to immune regulation and spatial immune organization, including regulatory T cells (#8) and tertiary lymphoid structures (#1), emphasized the biological complexity of tumor–immune interactions.

Therapeutic-oriented clusters, such as docetaxel (#3) and PD-1-related immunotherapy (#9), demonstrated strong connectivity with mechanistic clusters, reflecting the integration of molecular insights into clinical treatment strategies. Notably, clusters focused on predictive biomarkers (#7), tumor microenvironment (#5), and response prediction (#4) occupied intermediary positions between mechanism- and therapy-centered clusters, underscoring their translational relevance. The high modularity and silhouette values confirm the robustness of this clustering structure, collectively illustrating a coherent mechanism-to-therapy-to-translation knowledge framework in NSCLC research.

### 2.11. Temporal Evolution of Intellectual Clusters in NSCLC Translational Research

**Figure 9:**
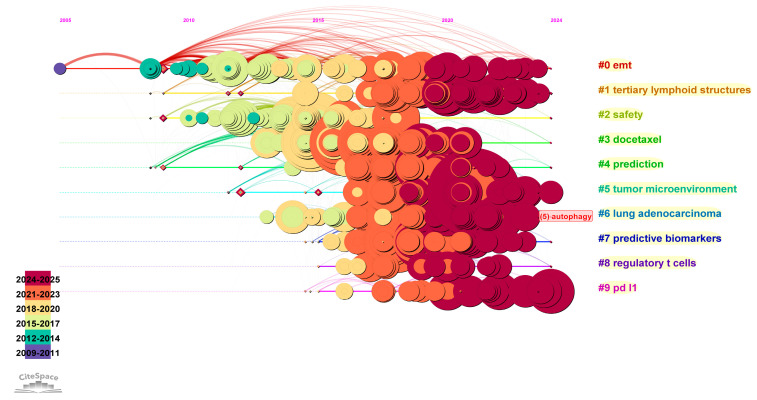
CiteSpace timeline visualization of co-citation clusters in NSCLC translational research (2009–2025). Each horizontal line represents a major cluster labeled by log-likelihood ratio terms, with node size proportional to citation frequency and color indicating publication year. Visualization generated using CiteSpace (version 6.4.R2). Key message: The timeline analysis highlights the sustained centrality of EMT across multiple translational phases and illustrates the progressive expansion of immune-related and tumor microenvironment-focused research culminating in PD-1-centered immunotherapy dominance.

The CiteSpace timeline visualization further elucidated the temporal dynamics and continuity of major intellectual clusters underpinning NSCLC translational research from 2009 to 2025. The analysis demonstrated that epithelial–mesenchymal transition (EMT; cluster #0) emerged early and persisted across the entire study period, maintaining a central position with dense inter-cluster linkages. This temporal persistence underscores EMT as a core mechanistic axis connecting tumor plasticity, therapeutic resistance, and immune modulation throughout multiple translational phases (Figure 9).

Clusters associated with lung adenocarcinoma (#6) and docetaxel-based chemotherapy (#3) were more prominent during earlier periods, reflecting histology-specific investigations and cytotoxic treatment paradigms that dominated the pre-immunotherapy era. From approximately 2015 onward, clusters related to tumor microenvironment (#5) and predictive biomarkers (#7) expanded markedly, indicating a shift toward biomarker-driven stratification and microenvironment-focused therapeutic strategies.

In the later phases (2018–2025), immune-related clusters, particularly PD-1 signaling (#9), regulatory T cells (#8), and tertiary lymphoid structures (#1), showed substantial growth and dense temporal overlap, reflecting the maturation and consolidation of immunotherapy research. Concurrently, clusters related to prediction (#4) and safety (#2) became increasingly active, highlighting the transition from therapeutic development toward response prediction, toxicity assessment, and clinical optimization. Collectively, the timeline analysis reveals a coherent evolution from tumor-intrinsic mechanisms to immune-centered and clinically contextualized translational research in NSCLC.

The timeline illustrates the sustained centrality of epithelial–mesenchymal transition (EMT, #0), early prominence of chemotherapy and histology-specific clusters, and the progressive expansion of tumor microenvironment, predictive biomarker, and immune-related clusters, culminating in PD-1-centered immunotherapy dominance in recent years.

### 2.12. EMT Induction Is Associated with Increased Secretion of Immunoregulatory and Microenvironment-Related Factors

**Figure 10:**
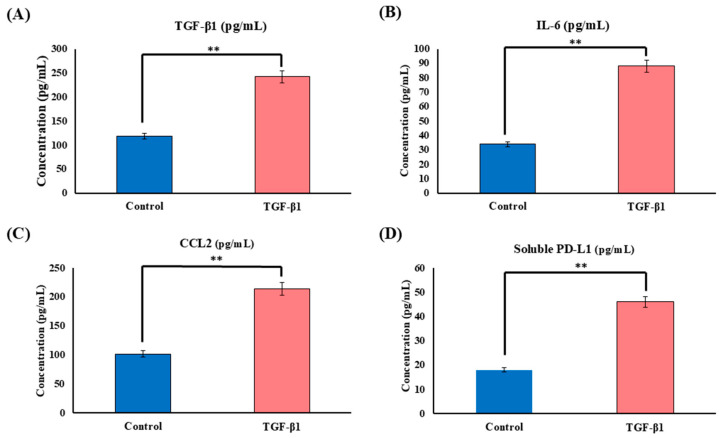
ELISA-based quantification of EMT-associated immunoregulatory mediators in TGF-β1-induced NSCLC cells. NSCLC cells were treated with TGF-β1 (5 ng/mL) for 48 h to induce epithelial–mesenchymal transition (EMT), and culture supernatants were subsequently analyzed by ELISA. (**A**) TGF-β1 concentration, (**B**) IL-6 concentration, (**C**) CCL2 concentration, and (**D**) soluble PD-L1 concentration. TGF-β1 treatment significantly increased the secretion of all measured mediators compared with untreated control cells, including TGF-β1 (~2.0-fold), IL-6 (~2.6-fold), CCL2 (~2.1-fold), and soluble PD-L1 (~2.6-fold). Data are presented as mean ± SD (n = 3). Statistical significance was determined using an unpaired Student’s *t*-test. ** *p* < 0.01 versus control.

An initial ELISA was conducted to experimentally validate the bibliometric identification of epithelial–mesenchymal transition (EMT) as a central mechanistic hub linking tumor biology with therapeutic and immune-related processes. Quantification of conditioned media from TGF-β1-treated NSCLC cells revealed significantly elevated levels of key microenvironmental and immunoregulatory mediators compared with untreated controls (Figure 10). Specifically, TGF-β1 levels increased from approximately 118 pg/mL to 242 pg/mL (~2.05-fold increase), IL-6 from 34 pg/mL to 88 pg/mL (~2.59-fold increase), and CCL2 from 102 pg/mL to 214 pg/mL (~2.10-fold increase) (*p* < 0.01 for all comparisons). These coordinated increases indicate the activation of an EMT-associated secretory phenotype characterized by enhanced inflammatory and stromal signaling. Notably, soluble PD-L1 levels also increased from 18 pg/mL to 46 pg/mL (~2.56-fold increase; *p* < 0.01), suggesting a functional linkage between EMT-associated signaling and immune checkpoint regulation.

Importantly, the purpose of the pilot ELISA validation was not to establish a novel biomarker discovery platform, but rather to experimentally support the bibliometric identification of EMT as a central mechanistic–translational hub in NSCLC research. The coordinated elevation of TGF-β1, IL-6, CCL2, and PD-L1 under EMT-inducing conditions provides preliminary biological evidence linking EMT-associated tumor plasticity with inflammatory signaling, immune checkpoint regulation, and tumor microenvironment remodeling. These findings support the hypothesis generated from the bibliometric mapping that EMT functions as a mechanistic interface connecting therapeutic resistance and immune adaptation in NSCLC.

## 3. Discussion

The present study provides a knowledge-depth, mechanism-informed mapping of translational NSCLC research, aligning with the scope of the IJMS Special Issue “Advances in Lung Research: From Mechanisms to Therapeutic Innovation”. While bibliometric approaches are often perceived as descriptive, the current analysis was deliberately designed to go beyond performance profiling by reconstructing the conceptual and intellectual architecture of NSCLC translational research—specifically the pathway through which mechanistic discoveries evolve into therapeutic modalities and ultimately into clinically relevant optimization strategies. By integrating co-word structure, thematic development, thematic evolution, and co-citation clustering with timeline visualization, this study captures not only “what has been published”, but also how mechanistic knowledge is organized and how it migrates toward therapeutic innovation, thereby producing an interpretive framework of high value for researchers, clinicians, and translational investigators.

The bibliometric evolution identified in this study closely parallels the clinical transformation of NSCLC management. Early mechanistic discoveries involving EGFR mutations directly enabled the development of EGFR tyrosine kinase inhibitors (TKIs), including gefitinib, erlotinib, osimertinib, and other next-generation targeted therapies that substantially improved progression-free survival in molecularly selected patients. Similarly, the emergence of PD-1/PD-L1 signaling as a dominant thematic cluster corresponds to the rapid integration of immune checkpoint inhibitors such as nivolumab, pembrolizumab, atezolizumab, and durvalumab into first-line and maintenance treatment strategies. Furthermore, EMT-related resistance mechanisms identified in the thematic evolution and co-citation analyses reflect growing translational efforts to overcome therapeutic resistance through combination immunotherapy, TGF-β pathway modulation, and tumor microenvironment-targeted approaches.

Annual scientific production demonstrated a pronounced acceleration in NSCLC translational research after 2018, consistent with a broader shift in lung cancer treatment paradigms [25]. This expansion is not simply a growth in publication numbers but reflects the increasing complexity of NSCLC biology and the urgent need for mechanism-based therapeutic innovation. Importantly, the steep rise in outputs from 2020 onward coincides with intensified research in immune checkpoint blockade, tumor microenvironment remodeling, and therapy combinations. Such growth patterns provide quantitative confirmation that lung cancer has become one of the most dynamic platforms in modern translational medicine—particularly suited for mechanism-to-therapy frameworks. In this context, our results mirror clinical reality: NSCLC management increasingly depends on mechanistic stratification and adaptive therapy selection rather than uniform treatment regimens. The author performance analysis further revealed that a relatively small group of highly productive investigators contributes substantially to the intellectual development of translational NSCLC research. Notably, many of these influential authors are affiliated with major research-intensive institutions identified in the institutional performance analysis, reflecting the concentration of expertise within leading oncology research centers. In addition, the strong international collaboration patterns observed in the global collaboration network further suggest that advances in NSCLC translational research are increasingly driven by interconnected scientific communities and cross-institutional partnerships.

Across keyword co-occurrence and thematic mapping, immunotherapy and tumor microenvironment emerged as dominant motor themes, reflecting high centrality (field relevance) and high density (theme maturity). This is a critical insight where it demonstrates that NSCLC therapeutic innovation has transitioned from a tumor-centric model toward an immune ecosystem model in which microenvironmental regulation dictates therapeutic response. In mechanistic terms, this shift emphasizes immune escape, antigen presentation dynamics, immune checkpoint signaling, and stromal–immune interactions as central determinants of NSCLC progression and treatment outcomes.

The clustering of PD-1/PD-L1-related concepts within the most developed and most relevant thematic region further indicates that immune checkpoint blockade is not merely a therapeutic endpoint but a mechanistic axis that now organizes large parts of lung cancer research. This finding aligns with the Special Issue’s objective to connect mechanistic advances in lung biology with therapeutic innovation, highlighting that NSCLC immunotherapy is best understood as a “mechanism-to-treatment continuum” rather than a drug category alone.

One of the most valuable contributions of this study is the identification of epithelial–mesenchymal transition (EMT) as a persistent intellectual and mechanistic hub. In CiteSpace co-citation clustering, EMT constituted the largest and most central cluster and displayed strong interconnections with microenvironmental, immune regulation, and therapeutic clusters. EMT has long been recognized as a driver of invasion and metastasis [25]; however, its prominence across translational clustering suggests a broader role as a mechanistic bridge connecting tumor plasticity to therapy resistance and immune suppression. In clinical terms, EMT-associated programs can shape immune infiltration, alter antigenicity, promote immune evasion, and modulate checkpoint signaling—mechanisms that directly influence immunotherapy responsiveness. EMT has increasing translational relevance because it contributes not only to invasion and metastatic dissemination, but also to therapeutic refractoriness across both targeted therapy and immunotherapy settings. EMT-associated signaling can promote EGFR-TKI resistance, immune exclusion, stromal remodeling, and PD-L1 upregulation, thereby reducing treatment responsiveness and facilitating adaptive tumor survival. The persistent centrality of EMT across the thematic evolution and CiteSpace timeline analyses suggests that EMT remains a mechanistic convergence point linking tumor plasticity with immune-regulatory adaptation. This may explain the growing interest in combination strategies integrating immune checkpoint blockade with TGF-β inhibition, stromal targeting, or EMT-modulating interventions.

This EMT-centered organization supports the concept that NSCLC resistance is not solely due to genetic mutations (e.g., secondary EGFR alterations) but is deeply tied to phenotypic plasticity and microenvironment-driven adaptation. Thus, EMT’s persistence across the timeline highlights it as a target for next-generation therapeutic innovation, including rational combinations of immune checkpoint inhibitors with agents targeting plasticity pathways, stromal remodeling, or metabolic vulnerabilities.

Thematic evolution analysis revealed a coherent translational trajectory across four mechanistic eras. The early period (2009–2013) was dominated by EGFR-TKI-related themes, including erlotinib and gefitinib, reflecting precision oncology’s initial phase centered on driver oncogene inhibition and drug sensitivity. This was followed by mechanistic consolidation during 2014–2018, where EMT emerged as an organizing concept linking resistance, progression, and therapy failure.

From 2019 onwards, immunotherapy and tumor microenvironment themes expanded and consolidated, indicating that the translational frontier migrated from tumor-intrinsic mechanisms toward immune-centric biology and clinical immunotherapy optimization. Notably, the appearance and strengthening of predictive biomarker and response prediction themes across later stages underscores how modern NSCLC therapy is increasingly guided by biomarker evaluation and immune profiling. In other words, translation in NSCLC is no longer limited to drug development but increasingly represents a diagnostic–therapeutic ecosystem involving predictive signatures, molecular stratification, and clinical decision support.

This progression illustrates a key message highly relevant to the Special Issue: in lung research, the most impactful therapeutic advances are those that integrate mechanistic insights with clinically implementable biomarkers and treatment strategies.

Beyond PD-1/PD-L1, the CiteSpace clusters for regulatory T cells and tertiary lymphoid structures provide deeper mechanistic insight into immune regulation and spatial immune organization. These themes signify that NSCLC immunotherapy innovation is being reshaped by spatial and cellular immune architecture, not only by checkpoint inhibition. Regulatory T cells influence immune suppression and therapeutic failure, while tertiary lymphoid structures have been linked to local anti-tumor immunity and response durability.

The emergence and growth of these clusters in later years suggests that the field is progressing from “single checkpoint blockade” toward complex immunomodulation strategies, including microenvironment reprogramming, stromal targeting, and immune niche engineering. This has clear therapeutic implications and supports the future direction of NSCLC innovation: enhancing immune infiltration, restoring effective antigen-driven T-cell activity, and overcoming microenvironment-mediated resistance.

An important and clinically meaningful finding is the emergence of prediction and safety clusters. Their growth indicates that NSCLC translational research is increasingly entering a late-phase innovation stage where therapeutic strategies must be refined in real-world contexts—balancing efficacy, toxicity, patient selection, and treatment sequencing. The rise in prediction and biomarker clusters illustrates that translational progress is not only driven by mechanistic discovery, but also by implementing mechanisms into predictive models and clinically meaningful stratification tools.

This is particularly relevant in immunotherapy, where response variability and immune-related adverse events require predictive tools that integrate tumor biology with host immune status. Consequently, our mapping supports the view that NSCLC translational research is now defined by the convergence of mechanistic biology with personalized clinical decision-making.

Bradford’s Law analysis revealed a concentrated set of core sources, indicating that translational NSCLC knowledge is disseminated through a relatively small number of high-impact oncology and immunology journals. This reflects the interdisciplinary nature of NSCLC innovation, positioned at the interface of tumor biology, immunology, and clinical oncology. The three-field plot further demonstrated tight linkages between seminal reference works, influential authors, and dominant translational themes. Such intellectual anchoring suggests that the field is driven by foundational clinical and mechanistic studies that continue to shape contemporary research priorities.

Importantly, this structure reinforces the argument that the present study is not merely bibliometric: it reconstructs how scientific knowledge is built, validated, and propagated within NSCLC translational medicine. By revealing the citation-driven backbone of the field, the study provides a roadmap for researchers seeking high-impact mechanistic questions, clinically relevant themes, and influential research lines.

The present knowledge-depth map has direct implications for translational lung research. First, it identifies EMT, tumor microenvironment biology, and immune regulation as mechanistic pillars that connect biology to therapy innovation. Second, it highlights predictive biomarkers and response prediction as increasingly mature translational priorities, emphasizing the need for multidimensional biomarkers integrating tumor genetics, immune composition, and microenvironmental context. Third, the mapping supports future innovation trajectories toward combination therapy strategies that target plasticity, stromal remodeling, immune suppression, and spatial immune architecture. Additionally, the translational themes identified in this study are closely associated with clinically meaningful improvements in patient outcomes. The incorporation of EGFR-targeted therapies and immune checkpoint inhibitors has substantially improved progression-free and overall survival in selected NSCLC populations compared with historical chemotherapy-based regimens. Nevertheless, acquired resistance, heterogeneous PD-L1 responsiveness, and tumor microenvironment-mediated immune escape remain major clinical challenges, explaining the persistent prominence of EMT, resistance biology, and predictive biomarker clusters throughout the knowledge structure identified in this analysis.

In addition to the bibliometric analysis, a pilot ELISA-based validation demonstrated that EMT induction is associated with elevated secretion of key immunoregulatory and tumor microenvironment-related mediators, including TGF-β1, IL-6, CCL2, and PD-L1. The selection of the NSCLC cell model (A549) is particularly relevant, as it represents a well-characterized epithelial-like lung adenocarcinoma system with high responsiveness to TGF-β-induced EMT [26,27], making it suitable for studying tumor plasticity and its downstream signaling consequences. Furthermore, the biomarkers assessed in this pilot study were deliberately chosen to reflect distinct but interconnected components of the EMT–immune–microenvironment axis. TGF-β1 serves as a canonical driver of EMT and a key regulator of tumor progression [25]; IL-6 represents a central pro-inflammatory cytokine implicated in tumor-promoting signaling and immune modulation [28]; CCL2 is critically involved in monocyte/macrophage recruitment and stromal remodeling [29]; and PD-L1 functions as a major immune checkpoint molecule directly linked to immunotherapy response [30].

The coordinated upregulation of these mediators under EMT-inducing conditions provides mechanistic evidence supporting the bibliometric identification of EMT as a central hub connecting tumor biology with immune regulation and therapeutic resistance. Importantly, the observed coupling between EMT signaling and immune checkpoint-associated pathways reinforces the concept that tumor plasticity actively contributes to immune evasion and reduced therapeutic responsiveness. Thus, this experimental validation strengthens the translational interpretation of the bibliometric findings by demonstrating that EMT is not only a conceptual node within the knowledge network but also a functionally relevant biological process driving immunoregulatory dynamics with direct clinical implications.

### Strengths and Limitations

An important strength of this study is the combination of complementary bibliometric and initial experimental strategies. Bibliometrix allowed for higher resolution keyword mapping, thematic evolution and performance profiling, whereas CiteSpace provided enhanced validation of the intellectual structure across a variety of robust co-citation clustering with substantial modularity and silhouette metrics—further supporting both reliability and interpretability of the knowledge base framework. Notably, this initial biological confirmation of suggested mechanistic–translational links—including a possible role for EMT in colocalizing immunoregulatory and TME signaling—draws on pilot ELISA validations. While still descriptive relative to the significant strength of bibliometrics, this analytical approach takes a first step from identifying broad trends toward an integration between mechanism and therapy. The rigorous search strategy, using title, abstract and author keywords, tailored it to the core that was clearly translational (and not just with a pure focus on diagnostic imaging studies), allowing a specific corpus that reflects translational NSCLC research.

However, some limitations need to be addressed. The dataset was obtained from the Web of Science Core Collection (SCI-Expanded) that did not cover all relevant NSCLC literature indexed uniquely in other databases. Since bibliometric analyses rely on metadata quality, the standardization of keyword assignments and citation dynamics [3], they may fail to account for emerging or newly developed topics. Additionally, the experimental validation we provide is more exploratory and only moderately informative; as such it should be viewed as preliminary support rather than definitive mechanistic confirmation. Despite these caveats however, the combination of quantitative mapping and rigorous experimental validation contributes to providing additional translational relevance and conceptual robustness to their findings.

## 4. Materials and Methods

### 4.1. Study Design

This study employed an integrative bibliometric, scientometric, and experimental framework to map the mechanism-to-therapy translational landscape in NSCLC. A combined analytical strategy was implemented to (i) quantify annual scientific production and identify core dissemination channels, (ii) characterize author productivity and intellectual linkages among key references, authors, and research themes, and (iii) reconstruct thematic structure and temporal evolution through keyword co-occurrence analysis and co-citation clustering. Bibliometric analyses were primarily conducted using the Bibliometrix package (R) (version 5.0) [31], while CiteSpace (version 6.4.R2) [32] was applied to validate the intellectual structure through co-citation clustering and timeline visualization.

**Figure 11:**
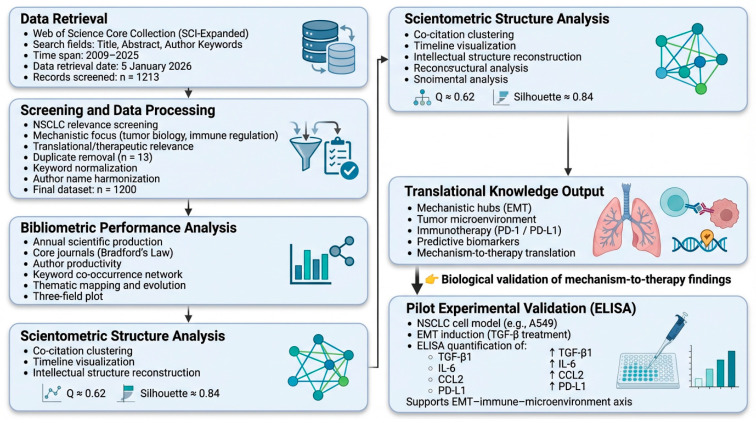
Illustration of the integrated workflow for translational mapping of NSCLC research. Bibliometric and scientometric analyses were performed using data retrieved from the Web of Science Core Collection, followed by keyword mapping, thematic evolution, and co-citation clustering. Translational knowledge outputs, including EMT, tumor microenvironment, and immunotherapy pathways, were further supported by pilot ELISA-based experimental validation, demonstrating increased secretion of immunoregulatory mediators under EMT-inducing conditions.

To extend beyond a descriptive mapping approach, a pilot experimental validation was incorporated to provide preliminary biological support for key mechanistic–translational relationships identified in the analysis. Specifically, an ELISA-based assay was performed in an NSCLC cell model to evaluate the secretion of EMT-associated immunoregulatory mediators, including TGF-β1, IL-6, CCL2, and PD-L1, under EMT-inducing conditions. This integrative approach enables the linkage of computationally derived knowledge structures with experimentally supported biological processes, strengthening the translational relevance of the findings. The overall study workflow and analytical framework, including the experimental validation component, are illustrated in Figure 11.

### 4.2. Data Source and Search Strategy

Bibliographic records were retrieved from the Web of Science Core Collection (WoSCC), limited to the Science Citation Index Expanded (SCI-Expanded) to ensure high-quality indexing and consistent citation metadata. The complete search query used for data retrieval from the Web of Science Core Collection (WoSCC), limited to the Science Citation Index Expanded (SCI-Expanded), is provided in the Supplementary Materials.

The search was limited to the Title (TI), Abstract (AB), and Author Keywords (AK) fields to ensure that retrieved records contained explicit mechanistic and translational content. The time span was restricted to 2009–2025, and results were further limited to English-language Articles indexed in the SCI-Expanded. All records were exported with full metadata and cited references for subsequent bibliometric analysis. The search was conducted on 5 January 2026.

### 4.3. Eligibility Criteria and Data Cleaning

All retrieved records were screened using predefined inclusion criteria: (i) NSCLC-focused studies, (ii) mechanistic content relevant to tumor biology and/or immune regulation, and (iii) explicit therapeutic or translational relevance (e.g., immunotherapy, chemotherapy, targeted therapy, biomarkers, response prediction, resistance, or safety).

A total of 1213 records were initially retrieved. Data cleaning procedures were performed prior to analysis to improve metadata consistency. Keyword standardization was performed prior to network construction to reduce semantic redundancy and improve thematic coherence. A manual thesaurus-based harmonization approach was applied using the Bibliometrix framework. Synonymous terms, abbreviations, spelling variants, and plural/singular forms were merged into standardized descriptors. Representative examples include the consolidation of “NSCLC” and “non-small cell lung cancer,” “PD-1” and “PD1,” and “tumor microenvironment” and “TME.” Similar normalization was applied to therapy-related and mechanistic terms where appropriate. The keyword harmonization list used for the analysis is provided in Supplementary File (Table S1) to facilitate reproducibility. After cleaning and eligibility filtering, 1200 records were retained for the final analyses.

### 4.4. Bibliometric Performance Analysis

Descriptive bibliometric indicators were computed using Bibliometrix (R) to characterize the growth and structure of NSCLC translational research. Annual scientific production was assessed to quantify temporal publication trends. The dispersion and concentration of research outputs across journals were evaluated using Bradford’s Law, identifying core sources responsible for a disproportionate share of publications. Author productivity was assessed using “most relevant authors” outputs, and author-level influence was evaluated using bibliometric impact indices (e.g., H-index) computed from the retrieved dataset.

### 4.5. Three-Field Plot Analysis (References–Authors–Keywords)

To examine the intellectual and conceptual structure connecting seminal studies, prolific researchers, and dominant themes, a three-field plot was generated linking cited references (CR), authors (AU), and merged keywords (KW_Merged). This approach enabled visualization of knowledge flow across foundational reference works, key contributors, and translational themes such as immunotherapy, tumor microenvironment, resistance mechanisms, and biomarker-guided therapy.

### 4.6. Keyword Co-Occurrence Network and Conceptual Structure Mapping

A keyword co-occurrence network was constructed to identify major thematic clusters and translational linkages within NSCLC research. Author keywords and Keywords Plus were consolidated using the cleaned thesaurus mapping. Co-occurrence edges were computed based on keyword co-appearance in the same documents, and network visualization was performed using standard Bibliometrix network plotting functions. Clusters were interpreted as mechanistic, translational bridge, or therapeutic/clinical domains based on dominant terms and network topology.

### 4.7. Thematic Mapping and Thematic Evolution

The conceptual development and maturity of research themes were evaluated using thematic mapping, which position clusters according to centrality (relevance/connectedness) and density (development/maturity). Themes were classified into four quadrants: motor themes, niche themes, basic themes, and emerging/declining themes.

To capture temporal translation dynamics, thematic evolution analysis was conducted across four time slices reflecting major translational phases in NSCLC research: 2009–2013, 2014–2018, 2019–2024, and 2025. Sankey-style evolution plots were used to visualize how dominant topics transitioned from early targeted therapy and sensitivity research to mechanistic consolidation and subsequently to immunotherapy and microenvironment-driven strategies.

### 4.8. CiteSpace Co-Citation Clustering and Timeline Visualization

To validate the intellectual structure derived from keyword-based analyses, co-citation network clustering was performed using CiteSpace (version 6.4.R2). Records were imported with cited references, and a time-sliced co-citation network was generated across 2009–2025. The clustering solution was evaluated using standard CiteSpace quality indicators, including modularity (Q) and mean silhouette score (S). Cluster labeling was conducted using log-likelihood ratio (LLR) terms to enhance interpretability.

In addition, a CiteSpace timeline visualization was constructed to examine the persistence and emergence of clusters over time, highlighting long-standing mechanistic hubs (e.g., epithelial–mesenchymal transition) and the later dominance of immune and translational clusters (e.g., PD-1, tumor microenvironment, predictive biomarkers, safety/prediction).

### 4.9. Software and Visualization

Analyses were conducted using R (Bibliometrix/Biblioshiny environment) for bibliometric computation, mapping, and thematic analyses, and CiteSpace for co-citation clustering and timeline visualization. All figures were generated from Bibliometrix and CiteSpace outputs, and final visualizations were exported in high resolution for publication. All figures were exported in high-resolution format (minimum 600 dpi) and optimized for readability by enlarging label fonts and adjusting layout spacing to ensure clarity in both digital and print formats.

### 4.10. Pilot ELISA Validation of EMT-Associated Immunoregulatory Signatures

To provide preliminary experimental support for the bibliometric findings, a pilot in vitro analysis was performed to evaluate the relationship between EMT and immunoregulatory signaling in NSCLC cells.

### 4.11. Cell Culture, Reagents and EMT Induction

A549 cell line (NSCLC cells) was obtained from College of Science, King Faisal University, and cultured in Dulbecco’s Modified Eagle Medium (DMEM) supplemented with 10% fetal bovine serum (FBS) and 1% penicillin–streptomycin under standard conditions (37 °C, 5% CO_2_). Recombinant human transforming growth factor-β1 (TGF-β1) used for EMT induction was obtained from R&D Systems (Minneapolis, MN, USA). Commercial enzyme-linked immunosorbent assay (ELISA) kits for the quantification of TGF-β1, interleukin-6 (IL-6), C-C motif chemokine ligand 2 (CCL2), and soluble Programmed Death-Ligand 1 (PD-L1) were purchased from Thermo Fisher Scientific (Waltham, MA, USA). All assays were performed in accordance with the manufacturers’ instructions, including recommended standards, reagents, and detection protocols. Cell culture reagents, including DMEM, FBS, and antibiotics (penicillin–streptomycin), were obtained from Gibco (Thermo Fisher Scientific, Waltham, MA, USA). EMT was induced by treatment with TGF-β1; 5 ng/mL for 48 h. Control cells were cultured under identical conditions without TGF-β1.

### 4.12. Collection of Conditioned Media

Following treatment, culture supernatants were collected, centrifuged at 1500× *g* for 10 min to remove cellular debris, and stored at −80 °C until analysis.

### 4.13. ELISA Quantification

The concentrations of TGF-β1, interleukin-6 (IL-6), CCL2, and soluble PD-L1 in the conditioned media were quantified using commercially available enzyme-linked immunosorbent assay (ELISA) kits according to the manufacturers’ protocols. All samples were analyzed in triplicate.

### 4.14. Statistical Analysis

Data were expressed as mean ± standard deviation (SD). Statistical comparisons between groups were performed using unpaired Student’s *t*-test. A value of *p* < 0.05 was considered statistically significant.

## 5. Conclusions

In conclusion, this study provides a comprehensive and mechanistically grounded translational knowledge map of NSCLC research integrating bibliometric, thematic, co-citation, and preliminary experimental validation approaches. The findings demonstrate that epithelial–mesenchymal transition (EMT) functions as a persistent mechanistic hub bridging tumor plasticity, therapeutic resistance, immune regulation, and tumor microenvironment remodeling, while tumor microenvironment- and PD-1-centered immunotherapy represent dominant motor themes driving recent therapeutic innovation. The temporal evolution further reveals a coherent transition from EGFR-TKI sensitivity paradigms toward immune-based, biomarker-guided, and microenvironment-oriented therapeutic strategies. Importantly, the pilot ELISA-based validation provided preliminary biological support for the proposed EMT-centered translational framework through the coordinated upregulation of TGF-β1, IL-6, CCL2, and PD-L1 under EMT-inducing conditions, reinforcing the mechanistic links identified through the bibliometric analyses. Collectively, these findings position the present work as a knowledge-depth translational analysis extending beyond descriptive bibliometrics, offering mechanistically informed and clinically relevant insights for advancing NSCLC therapeutic innovation from molecular discovery toward translational and precision oncology applications.

## Figures and Tables

**Table 1:** Top 10 Relevant Authors with Institutional Affiliation, Publication Output and Global H-index.

No.	Full Author Name	Institution	Research Area	No. of Documents (Dataset)	Global H-Index *
1	Jing Wang	Tongji University	NSCLC immunotherapy, PD-1/PD-L1	40	~70
2	Hongbin Chen	Tongji University School of Medicine	Immune checkpoint therapy, tumor microenvironment	37	~55
3	Li Zhang	Sun Yat-sen University Cancer Center	Lung cancer clinical trials	31	~85
4	Yi Yang	Southern Medical University	EMT and tumor progression	26	~65
5	Jian Li	Fudan University	Lung cancer targeted therapy	22	~60
6	Caicun Zhou	Tongji University (Shanghai Pulmonary Hospital)	EGFR-TKI therapy, immunotherapy trials	20	~110
7	Jing Wan	Tongji University	Thoracic oncology translational research	19	~70
8	Shun Lu	Shanghai Jiao Tong University	NSCLC targeted therapy	16	~75
9	Yi-Long Wu	Guangdong Lung Cancer Institute/Southern Medical University	EGFR-mutant NSCLC therapy	16	~150
10	Zhong Zhao	Chinese Academy of Medical Sciences	Lung cancer molecular biomarkers	16	~50

* Global H-index values are approximate values derived from Google Scholar/Scopus profiles of leading researchers in lung cancer and thoracic oncology.

**Table 2:** Top 10 Relevant Institutional Affiliations Contributing to NSCLC Translational Research.

Number	Institution	Country	Number of Articles
1	Tongji University	China	206
2	University of Texas MD Anderson Cancer Center	USA	151
3	Sun Yat-sen University	China	140
4	Chinese Academy of Medical Sciences and Peking Union Medical College	China	125
5	Fudan University	China	124
6	Southern Medical University	China	89
7	Nanjing Medical University	China	84
8	Soochow University	China	82
9	Shanghai Jiao Tong University	China	81
10	Central South University	China	79

Article counts represent institutional affiliation frequency within the dataset (N = 1200). Publications with multiple institutional affiliations may be counted more than once.

## Data Availability

The bibliometric datasets analyzed in this study were derived from Web of Science Core Collection. Raw data and analysis scripts are available from the corresponding author upon reasonable request.

## Supplementary Materials

The following supporting information can be downloaded at: https://www.mdpi.com/article/10.3390/ijms27125245/s1.

## Abbreviations

The following abbreviations are used in this manuscript:

ADC	Antibody–Drug Conjugate
AK	Author Keywords
AU	Authors
CR	Cited References
DDR	DNA Damage Response
EGFR	Epidermal Growth Factor Receptor
EMT	Epithelial–Mesenchymal Transition
H-index	Hirsch Index
ICI	Immune Checkpoint Inhibitor
IJMS	International Journal of Molecular Sciences
KW	Keywords
KW_Merged	Merged Keywords
LLR	Log-Likelihood
