# Supplementary material for: From Mechanisms to Therapeutic Innovation in Non-Small Cell Lung Cancer: A Knowledge-Depth Translational Mapping with Experimental Validation
Source: Int J Mol Sci. 2026 Jun 10;27(12):5245. doi: 10.3390/ijms27125245 (PMC13299734; doi:10.3390/ijms27125245)
Supplement: Supplementary file 1 [file ijms-27-05245-s001.zip › ijms-4290573-supplementary.pdf]

1. Supplementary File S1. Web of Science Search Strategy

Database: Web of Science Core Collection (SCI-Expanded)
Search fields: Title (TI), Abstract (AB), Author Keywords (AK)
Timespan: 2009–2025
Language: English
Document types: Article
Search date: 5 January 2026

```
(
 (TI=("non-small cell lung cancer" OR NSCLC) OR AB=("non-small cell lung cancer" OR NSCLC) OR AK=("non-small cell lung cancer" OR NSCLC))
 AND
 (TI=("tumor microenvironment" OR TME OR "immune evasion" OR "DNA damage response" OR DDR OR ferroptos* OR senescen* OR EMT)
  OR AB=("tumor microenvironment" OR TME OR "immune evasion" OR "DNA damage response" OR DDR OR ferroptos* OR senescen* OR EMT)
  OR AK=("tumor microenvironment" OR TME OR "immune evasion" OR "DNA damage response" OR DDR OR ferroptos* OR senescen* OR EMT))
 AND
 (TI=(immunotherap* OR "immune checkpoint" OR "PD-1" OR "PD-L1" OR "targeted therapy" OR TKI OR ADC OR "antibody-drug conjugate" OR bispecific*)
  OR AB=(immunotherap* OR "immune checkpoint" OR "PD-1" OR "PD-L1" OR "targeted therapy" OR TKI OR ADC OR "antibody-drug conjugate" OR bispecific*)
  OR AK=(immunotherap* OR "immune checkpoint" OR "PD-1" OR "PD-L1" OR "targeted therapy" OR TKI OR ADC OR "antibody-drug conjugate" OR bispecific*))
 AND
 (TI=(translat* OR resistance OR biomarker* OR "clinical trial" OR phase OR patients)
  OR AB=(translat* OR resistance OR biomarker* OR "clinical trial" OR phase OR patients)
  OR AK=(translat* OR resistance OR biomarker* OR "clinical trial" OR phase OR patients))
)
AND PY=(2009-2025)
AND DT=(Article)
AND LA=(English)
NOT (TI=(radiomics OR PET OR "computed tomography" OR "deep learning") OR AB=(radiomics OR PET OR "computed tomography" OR "deep learning"))
```

2. Supplementary Table S1. Keyword Harmonization Thesaurus Used for Data Cleaning

Keyword normalization was performed to harmonize synonymous terms and abbreviations prior to bibliometric analysis.

Original Keyword Variants	Standardized Keyword
NSCLC; non-small cell lung cancer	Non-small cell lung cancer
PD1; PD-1	PD-1
PD-L1; PDL1	PD-L1
tumor microenvironment; TME	Tumor microenvironment
EMT; epithelial mesenchymal transition	Epithelial–mesenchymal transition
EGFR mutation; EGFR-mutant	EGFR mutation
immune checkpoint blockade; checkpoint inhibitor	Immunotherapy
targeted therapy; molecular targeted therapy	Targeted therapy
